# Functional gut variability drives metabolic and transcriptional features in neonatal piglets

**DOI:** 10.1038/s41538-025-00571-z

**Published:** 2025-10-16

**Authors:** Xuan He, Shannon Shoff, Hanna Lee, Merete Lindberg Hartvigsen, Anne Staudt Kvistgaard, Carolyn M. Slupsky

**Affiliations:** 1 Department of Nutrition, University of California-Davis, Davis, CA USA; 2 Department of Food Science and Technology, University of California-Davis, Davis, CA USA; 3 Arla Foods Ingredients Group P/S, Viby J, Denmark; 4 Present Address: Department of Food Science & Nutrition, Cal Poly, San Luis Obispo, CA USA

**Keywords:** Biochemistry, Metabolomics

## Abstract

The inherent inter-individual variability and associated complexity of dietary response constitute a major research challenge, but also present an opportunity to trace personalized patterns that may serve as targets for improving health. Here, we investigated the function of α-lactalbumin, a rich source of tryptophan, in a formula feeding study. We leveraged neonatal piglets as a preclinical model for human infants and took a systems-level approach that integrates evidence from serum, urine, liver, brain, and the gastrointestinal tract. Transcriptional and metabolomics analysis revealed an individualized, divergent response to α-lactalbumin linked to either efficient utilization of tryptophan by the host or production of indole-3-lactate by intestinal microbiota. This variability was further highlighted by differences in metabolic and immunological effects in a tissue-specific manner. Our work highlights the importance of considering the nutrition-microbiota-host metabolism axis to optimize the phenotypic response of a diet.

## Introduction

It is well established that early-life nutrition impacts long-term health^1^. Although human milk is generally regarded as the gold standard for early-life nutrition^2^, when breastfeeding is not an option, infant formula is the closest alternative to supply infants with the fundamental nutrients necessary to support growth and development. Despite many advances in infant formula composition and manufacturing, both the total protein content and protein composition of infant formulas still differ from human milk^3^. Emerging evidence suggests that formula-fed infants exhibit a different metabolic profile than breastfed infants, especially with respect to markers of amino acid metabolism. Elevated circulating branched-chain amino acids (BCAAs) are a hallmark of formula-fed infants, which are believed to stimulate the mechanistic target of rapamycin (mTOR) signaling pathway and may promote adipogenesis and increased risk of obesity later in life^4,5^. Given the emerging evidence of the long-lasting health implications of formula-feeding, research studies that aim to evaluate the optimal protein level and the protein composition are much needed to narrow the gap between human milk and infant formula.

A diverse and resilient gut microbiota provides essential health benefits to its host, including immune regulation, inhibition of pathogens, strengthening of the intestinal barrier, energy harvesting, vitamin production, and metabolism^6,7^. While diet offers an effective strategy for modulating gut microbial composition, the gut microbiota can also act as key mediators of host dietary response. Various studies have demonstrated that individuals with different microbiota profiles respond differently to the same dietary intervention^8–10^. Therefore, rather than focusing on the average impact of a dietary intervention, inter-individual variability and personalized response to diet should be considered. Yet, exactly how individual-specific gut ecology responds to diet, lifestyle, and environmental factors and the integrated effect on an individual’s health and metabolism remains to be elucidated. Although more studies are beginning to utilize data from gut microbiota to inform precision nutrition and guide diet-based strategies for disease prevention and treatment, the various lifestyles, dietary habits, and environmental factors of participants in human intervention studies are difficult to control and pose a significant challenge to understanding the complex mechanisms and interplay between diet, host, and gut microbiota.

Compared to other animal models, the domestic pig (*Sus scrofa*) more closely resembles human anatomy, digestive physiology, immunology, and metabolism; therefore, the pig serves as a valuable preclinical model to improve our understanding of digestion, absorption, and metabolic processes following a nutritional intervention^11–13^. Many studies have demonstrated the use of suckling piglets as a suitable translational model for researching the short and long-term functional impact of infant nutrition^14,15^. The recent development of the domestic pig reference genome assembly (*Sscrofa11.1*) and improvements to the genome functional annotation provide a valuable resource to bring new insights into understanding the molecular basis of human health and disease^16,17^.

To further advance our understanding of the personalized associations between the microbiome, nutrition, and metabolic health, we leveraged a neonatal piglet model to systematically characterize the complex interrelationship between diet and gut microbiota and their collective effects on host health and development. Complementing metabolomics data generated throughout the gastrointestinal tract (GIT) during the early feeding period, we further integrated quantitative serum, liver, brain, and urine metabolome data, as well as liver and brain transcriptome data, to investigate the metabolic consequences behind the differential responses to diet. Here, we demonstrated the use of neonatal piglets as a preclinical model to understand the functional implications of milk components on infant health through a systems biology approach with the aim of bringing translatable knowledge into applications that can be applied to human infants.

## Results

### Crosstalk between blood, liver, and brain, and the influence of diet

**Fig. 1:**
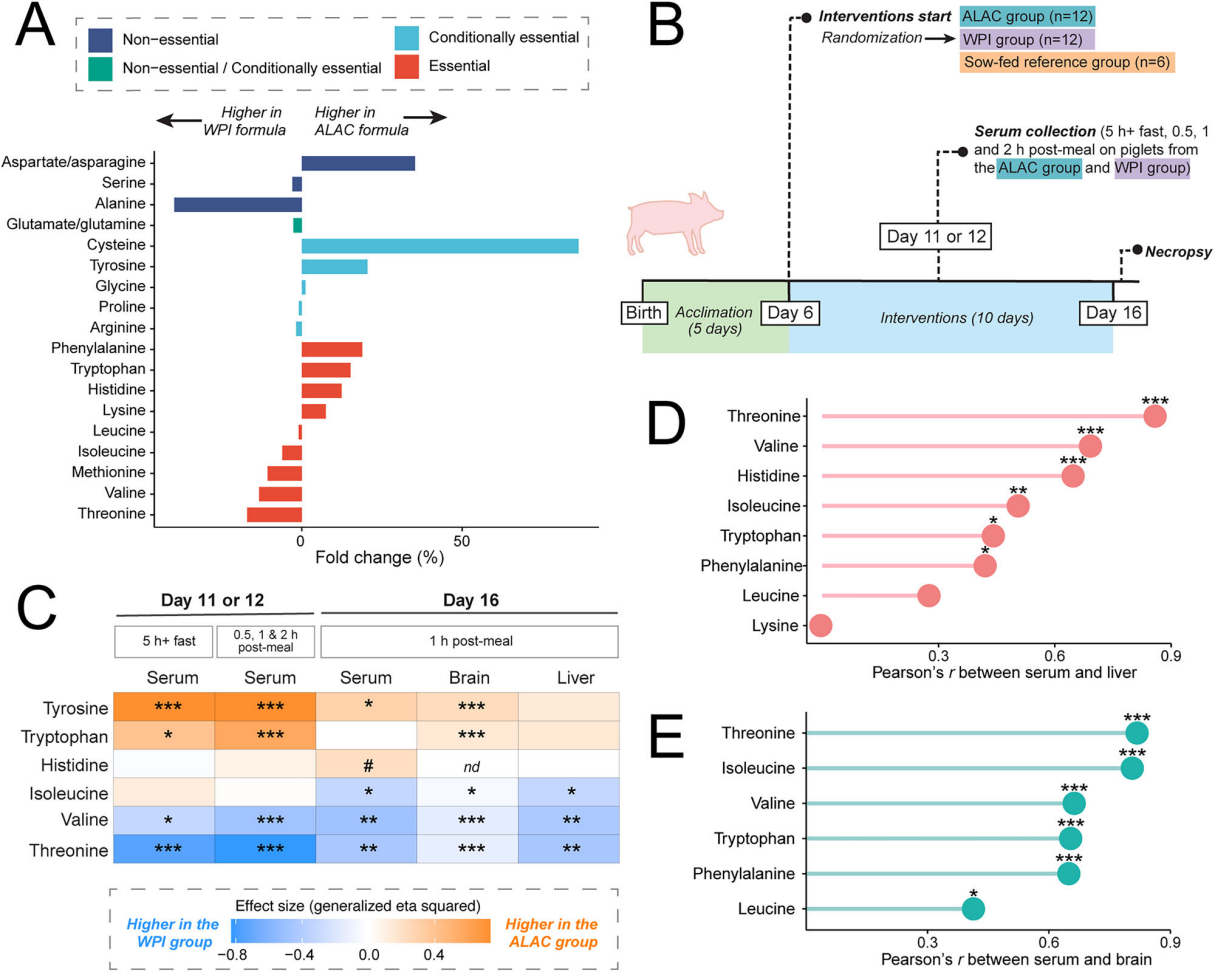
The effect of diet on neonatal metabolism. **A** Amino acid compositional difference between diets formulated using α-lactalbumin-enriched whey protein isolate (ALAC) and standard whey protein isolate (WPI). Detailed nutritional composition of formulas and sow milk is presented in Supplementary Tables 1 and 2. **B** Overview of the study design. At birth, 30 piglets were allowed to suckle from their sow until Day 6 and subsequently randomized to receive either the ALAC formula, *n* = 12, the WPI formula, *n* = 12, or remain with the sow (SF, *n* = 6). From Days 6–16, piglets in the ALAC and WPI groups were housed by group and bottle-fed formula to meet their needs in 8 equal portions. Piglets in the SF group remained with their birth sow for the duration of the study. On Day 7, one SF piglet was euthanized due to an accidental injury caused by the sow. Additionally, one female piglet from the ALAC group was reassigned to the sow-fed group due to unwillingness to consume the assigned formula. A total of 11 piglets from the ALAC group (6 males, 5 females), 12 piglets from the WPI group (6 males, 6 females), and 6 piglets from the SF group (3 males, 3 females) completed the study. On Days 11 and 12, postprandial venous blood was collected, and GC-MS-based untargeted metabolomics was performed on serum. On Day 16, piglets were anesthetized and sacrificed approximately 1 h after their last meal, and blood (from cardiac puncture), urine, and tissue samples were collected. ^1^H-NMR-based metabolomics was used to assess serum. **C** Differences in essential amino acid levels in serum, liver, and brain are linked to dietary levels. The differences between the ALAC group and WPI groups were evaluated using ANOVA followed by Benjamini & Hochberg FDR multiple comparison correction on generalized log-transformed data. Values in the heatmap are expressed as effect size (generalized eta squared). Confounding factors (litter, multiple time points, regions, formula volume, and time since meal) were accounted for in the model where necessary. **D**, **E** Pearson’s correlation illustrates a strong positive correlation between circulating, liver, and brain essential amino acid levels on Day 16. Data from all three groups were used for the analysis. Brain metabolite concentrations were computed as the average of the hypothalamus, hippocampus, prefrontal cortex, and striatum concentrations. *P*-values were corrected for multiple testing using the Benjamini & Hochberg FDR method. *** adjusted *p* < 0.001, ** adjusted *p* < 0.01, * adjusted *p* < 0.05, # unadjusted *p* < 0.05, *nd* not determined.

In this study, we developed and produced two isocaloric, liquid-based piglet formulas that matched the nutritional content of sow milk. The only difference between the two formulas was the source of whey protein: (i) bovine α-lactalbumin-enriched whey protein isolate (the ALAC formula) or (ii) standard bovine whey protein isolate high in β-lactoglobulin but low in α-lactalbumin (the WPI formula). A small amount of free amino acids (<8% of total amino acids) was formulated into the diets to prevent any negative experimental outcomes as a consequence of essential amino acid deficiency. After formula production, total protein and amino acid composition were quantified. As expected, the use of different proteins in the formulas resulted in differences in the dietary amino acid composition of each formula. The ALAC formula had higher levels of cysteine, phenylalanine, tryptophan, and histidine and lower levels of threonine, valine, methionine, and isoleucine compared to the WPI formula (Fig. 1A).

To mimic human formula-feeding practices, piglets stayed with their respective sow for 5 days after birth to allow consumption of colostrum. On Day 6, piglets were weight-, sex-, and litter-matched, and randomly assigned to either continue to consume the sow’s milk (the sow-fed reference group, SF) or consume one of the two piglet formulas, the ALAC formula or the WPI formula. To transition piglets from sow’s milk to formula, all formula-fed piglets were cuddled and held using a warm towel and hand-fed using nursing bottles every 3 h. This gentle handling by human caregivers also ensured the sharing and exchanging of environmental microbes during this critical developmental period. All formula-fed piglets were group-housed based on their assigned formula to minimize stress and ensure proper social behavior maturation. This dietary intervention continued for a total of 10 days, ending on Day 16, which is equivalent to approximately 1–2 months of a human’s age^13^. The study design is illustrated in Fig. 1B.

To examine the impact of whey protein source on infant metabolism, serum samples were collected on Day 11 or 12, and again on Day 16. Liver, urine, and four brain regions (hypothalamus, hippocampus, prefrontal cortex, striatum) were collected on Day 16. Serum metabolites from Day 11 or 12 were subjected to untargeted GC-MS analysis. Serum, urine, brain, and liver metabolites on Day 16 were quantified using a targeted NMR and GC-MS metabolomics approach. To explore the metabolic impact of diet, we chose the essential amino acids that differed most between the two formulas as the dietary signatures of each formula diet (ALAC formula: high in phenylalanine + tyrosine, tryptophan, and histidine; WPI formula: high in threonine, valine, and isoleucine). Although methionine and cysteine were quantified, because their metabolism differs between humans and pigs^18^, they were excluded from this analysis.

The observed differences in essential amino acid levels of each diet can be directly attributed to the differing amino acid compositions of the whey protein source used to develop each of the study formulas. These differences were correspondingly observed in the serum and liver metabolomes, except for serum tryptophan on Day 16 (Fig. 1C). Serum tryptophan was significantly higher in the SF piglets compared to their formula-fed counterparts; however, the difference between the two formula-fed groups was not significant (Supplementary Fig. 1A). Notably, the WPI formula contained a lower tryptophan level than the ALAC formula (WPI formula: 0.89 g/L, ALAC formula: 1.03 g/L), and both formulas had higher tryptophan levels compared to sow milk (0.66 g/L). This unexpected finding in circulating tryptophan further motivated us to explore the complex relationship between utilization efficiency and diet-induced metabolic response. At approximately 1 h after formula-feeding or sow milk letdown following oxytocin injection, the diet-induced change in circulating essential amino acids was correlated with hepatic and brain levels (Fig. 1D, E). We and others have previously demonstrated that human infants exhibit elevated blood levels of essential amino acids following formula-feeding^19,20^. This association between circulating, liver, and brain essential amino acids highlights the interconnectedness between systemic amino acid availability and organ-specific metabolism. Importantly, since essential amino acids are derived exclusively from the diet, these results clearly illustrate how dietary intake directly modulates the utilization of these nutrients in the metabolic pathways of the liver and brain.

### Variation of the metabolome along the gastrointestinal tract

**Fig. 2:**
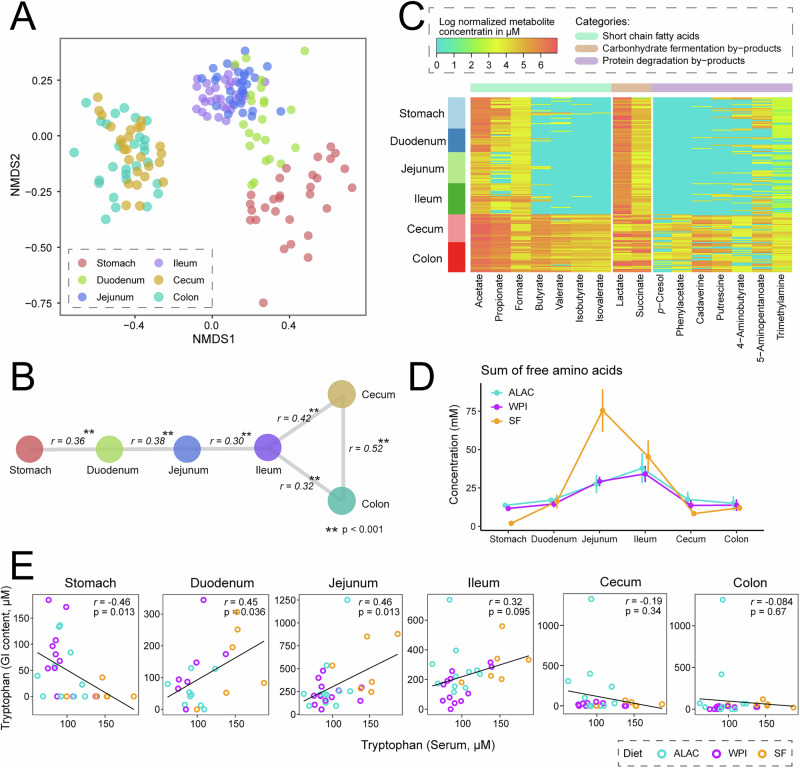
Quantitative metabolome profile of gastrointestinal contents using ^1^H-NMR. **A** Two-dimensional non-metric multidimensional scaling (NMDS) based on the Canberra distance of luminal contents from six different regions of the gastrointestinal tract reveals unique metabolic profiles based on anatomy (stress = 0.128). **B** Mantel test based on Canberra distance demonstrates connectivity between each gastrointestinal site. **C** Heatmap of microbial-derived by-products obtained from the stomach, duodenum, jejunum, ileum, cecal, and colon content illustrates possible microbial fermentation activity in the stomach and small intestine. **A**–**C** Data from all three groups were used for the analysis. **D** Sum of free amino acids that are solely derived from milk proteins demonstrates a difference between sow-fed and formula-fed piglets with respect to amino acid digestion and absorption efficiency. Data are represented as mean ± SEM. **E** Pairwise Pearson’s correlation relationship between serum tryptophan and tryptophan level throughout the gastrointestinal tract.

The gastrointestinal tract (GIT) is essential for metabolism, and the environment along the GIT is highly variable with regional specialization in nutrient digestion and absorption. To assess the impact of diet on digestion, we examined the luminal content of the stomach, duodenum, jejunum, ileum, cecum, and colon collected approximately 1 h after the last bottle feeding of piglets. Luminal contents from anatomically and functionally distinct tissues revealed unique metabolic profiles (Fig. 2A). The contents obtained from one GIT region were highly correlated to those collected from adjacent regions (Fig. 2B). The cecum and colon were found to be more similar to each other and distinct from other regions. Although anatomical location had the greatest impact on the metabolome profile overall, altering the whey protein source alone weakly contributed to the metabolic differences within the GIT (variance explained by region: 57.9%; variance explained by whey protein source: 1.1%; *p* < 0.01, pairwise PERMANOVA using the adonis function with Canberra distance).

Interestingly, investigating the distribution of microbial fermentation by-products along the GIT regions revealed that a few short-chain fatty acids (acetate, propionate, and formate), carbohydrate fermentation by-products (lactate and succinate), and protein degradation by-products (5-aminopentanoate and trimethylamine) were present in high levels in the upper GIT (Fig. 2C). This observation from the neonatal piglet model may serve as evidence to suggest the presence of active microbes in the upper GIT during the suckling period.

Furthermore, the total concentration of free amino acids measured in the digesta was highest in the small intestine and decreased progressively towards the lower GIT (Fig. 2D). This trend aligns with what is known about protein digestion and amino acid absorption. Notably, formula-fed piglets had significantly lower free amino acids in the small intestine compared to their sow-fed counterparts. This difference may be influenced by multiple factors, including variations in proteolytic activity, the bioavailability and digestibility of proteins, and the specific amino acid composition of the piglet diet.

To illustrate the utilization efficiency of tryptophan in sow milk and piglet formula, a pairwise correlation relationship between serum tryptophan and the tryptophan levels throughout the GIT was evaluated (Fig. 2E). Serum tryptophan was positively correlated with free tryptophan found in the duodenum, jejunum, and ileum, but not related to the tryptophan level collected from other GIT sites. Furthermore, this trend was strongly driven by feeding mode, as the formula-fed piglets showed significantly less free tryptophan in the jejunum (2-way ANOVA on generalized log-transformed data, *p* = 0.017, generalized eta squared = 0.209, litter effect was controlled). Therefore, although sow milk contains lower dietary tryptophan than the two formulas, the higher serum tryptophan in those piglets may be related to a more efficient release of tryptophan in the small intestine.

### Functional variability in gut microbial activity contributes to a unique diet-induced metabolic outcome

**Fig. 3:**
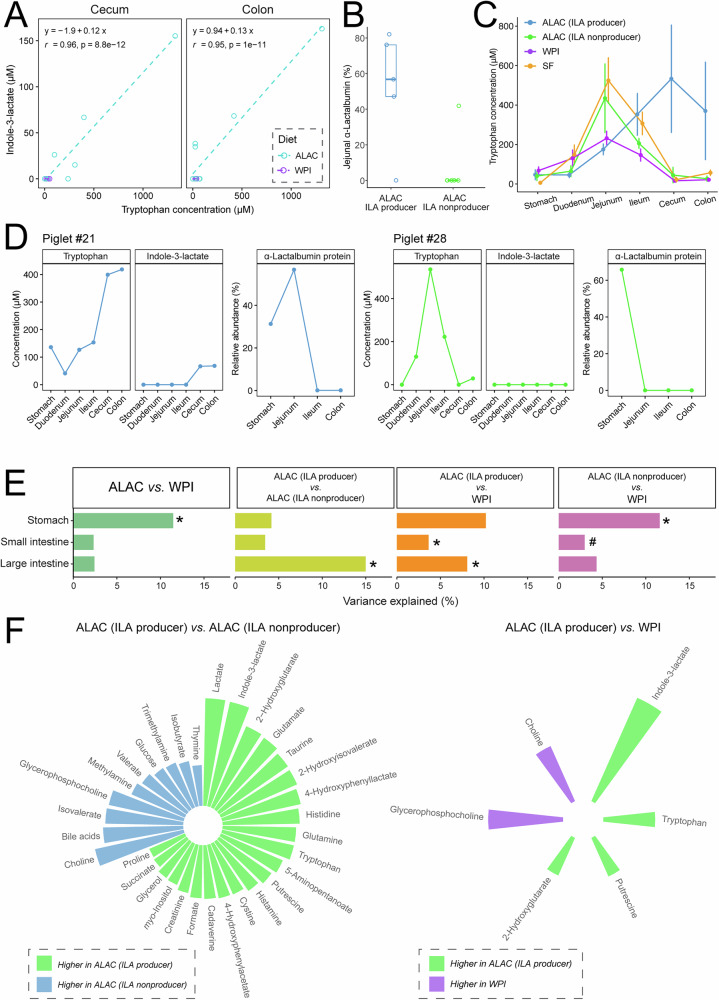
Significance of ILA-dependent phenotype on the metabolome. **A** Correlation between tryptophan and indole-3-lactate in the cecal and colon contents. The association coefficient was evaluated using Pearson’s correlation *r* and visualized via linear regression. **B** The relative abundance of intact α-lactalbumin in jejunal content. The relative intensity value (%) was quantified using normalized gel band intensity from SDS-PAGE at approximately 14 K molecular weight by total lane intensity. The corresponding gel image can be found in Supplementary Fig. 2. **C** Distribution of tryptophan throughout the gastrointestinal tract. Data are represented as mean ± SEM. **D** Concentration of tryptophan, indole-3-lactate, and relative abundance of intact α-lactalbumin of two sex- and weight-matched piglets from the same litter. The corresponding gel image can be found in Supplementary Fig. 2. **E** Permutational multivariate analysis of variance (PERMANOVA) using the adonis function with Canberra distance on logged metabolomics data after excluding indole-3-lactate. *P*-values from PERMANOVA were adjusted for multiple hypothesis testing using the Benjamini-Hochberg FDR procedure. * Adjusted *p*-value < 0.05, # unadjusted *p*-value < 0.05 (not significant after adjustment). **F** Significantly different metabolites in the cecal and colon contents between the ALAC ILA producers and the ALAC ILA non-producers (left), and between the ALAC ILA producers and the WPI group (right). Univariate analysis was performed using ANOVA on generalized log-transformed data with Benjamini & Hochberg FDR correction. Factors (region: colon or cecum, and litter) were controlled for in the model. The height of each bar represents the effect size (generalized eta squared after accounting for the effect of region and litter).

It is important to highlight that the majority of piglets had undetectable tryptophan in the cecum and colon, whereas a few ALAC piglets had a high level of tryptophan in these regions of the GIT (Fig. 2E), which was likely released from α-lactalbumin protein resistant to digestion in the small intestine. The unabsorbed tryptophan in the cecum and colon further positively correlated with the level of indole-3-lactate (ILA), a product of microbial tryptophan metabolism (Fig. 3A). Using targeted ^1^H-NMR metabolomics, we found that only 45% (5 out of 11) of the piglets fed the ALAC formula had quantifiable levels of ILA in the cecal and colon contents. In contrast, none of the piglets from the WPI and SF groups had detectable ILA in these regions. To understand the interplay between digestion efficiency and microbial activity on metabolism, piglets that received the ALAC formula were further categorized into two groups based on the ILA level in the cecum and colon: ILA non-producers (undetectable levels), or ILA producers (detectable levels (>10 μM)).

In comparison to the ILA non-producers, ILA producers were characterized by having higher levels of intact α-lactalbumin in the jejunum (Fig. 3B) and unabsorbed tryptophan in the cecum and colon (Fig. 3C). This difference was not due to genetics, as two piglets of the same sex, of similar weight, from the same litter, and reared in the same pen were dichotomously classified (Piglet #21 as an ILA producer and Piglet #28 as an ILA non-producer) (Fig. 3D). Even removing ILA from the cecal and colon metabolome data, the metabolic composition between the ALAC ILA producers and non-producers, or between the ALAC ILA producers and the WPI piglets revealed distinct differences (Fig. 3E), suggesting a unique microbial functional profile that is associated with ILA production. Under the same diet, ILA producers had significantly higher levels of lactate (Fig. 3F), which may indicate strong fermentation activity from lactic acid bacteria.

**Fig. 4:**
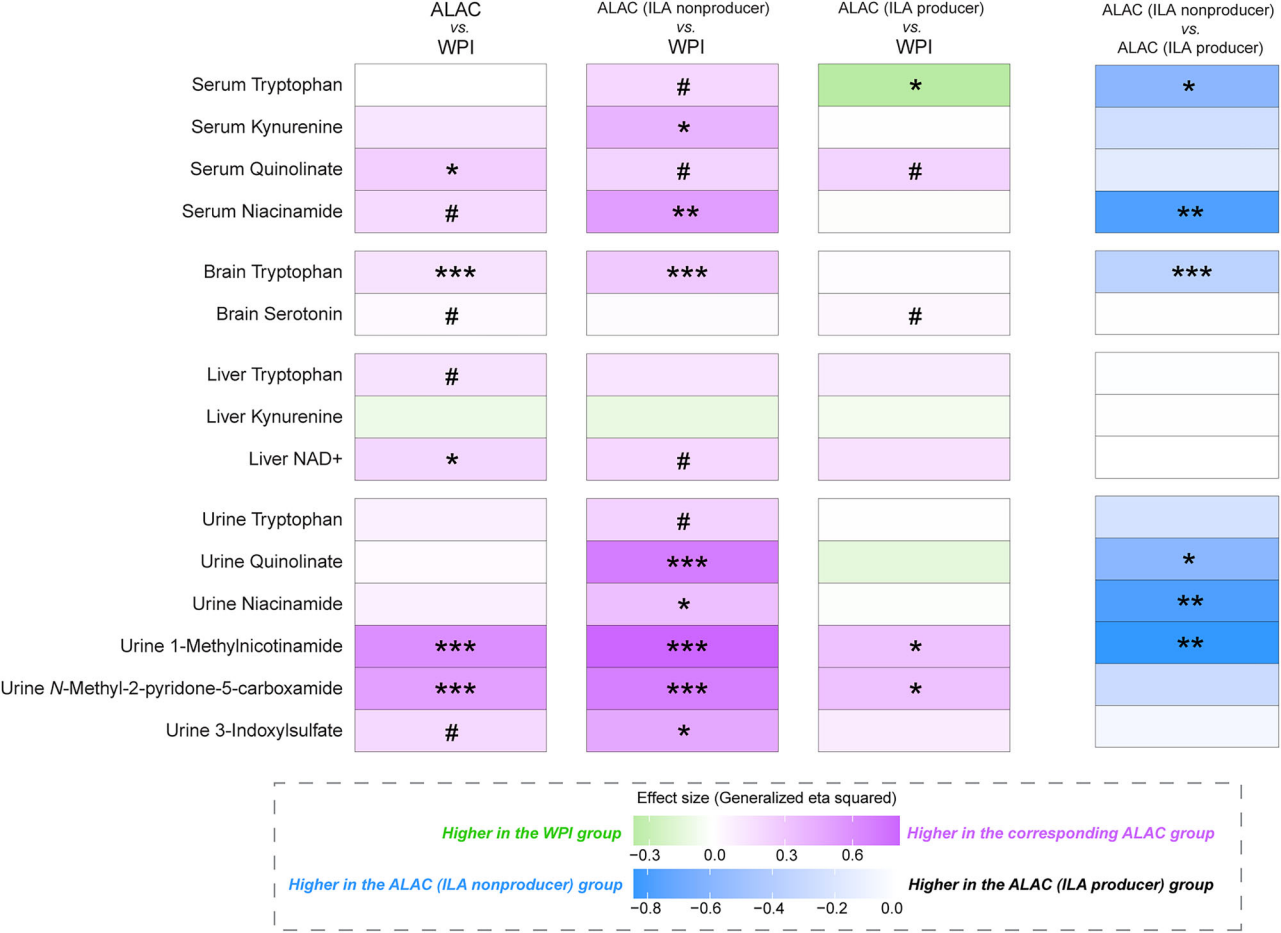
Host tryptophan metabolism interacts with microbial function of ILA production in the intestine. Serum was collected at necropsy at Day 16 at approximately 1 h post-meal. Measurements of tryptophan and kynurenine in serum and liver, measurements of tryptophan and serotonin in brain regions (prefrontal cortex, hippocampus, hypothalamus, and striatum) were determined via targeted GC-MS-based metabolomics. Measurements of serum quinolinate and niacinamide, liver NAD, as well as urinary levels of tryptophan, quinolinate, niacinamide, 1-methylnicotinamide, *N*-methyl-2-pyridone-5-carboxamide, and 3-indoxylsulfate were assessed via targeted ^1^H-NMR-based metabolomics. Values in the heatmap are expressed as effect size (generalized eta squared). Confounding factors (litter, multiple time points, formula volume, time since meal) were accounted for in the model when necessary. *p*-values are not adjusted for multiple comparisons. *** unadjusted *p* < 0.001, ** unadjusted *p* < 0.01, * unadjusted *p* < 0.05, # unadjusted *p* < 0.1.

Coincident with delayed tryptophan release in the GIT, ILA producers also appeared to have lower tryptophan in circulation compared to the ILA non-producers fed the same ALAC formula (Supplementary Fig. 1B). In contrast, ALAC ILA non-producer piglets exhibited a profound increase in metabolites that are a part of host tryptophan metabolism compared with the WPI piglets (Fig. 4). Notably, a few tryptophan metabolites including kynurenine, 3-indoxylsulfate, and ILA are known as key signaling molecules that trigger the aryl hydrocarbon receptor (AhR)-dependent signaling pathway^21^. As both kynurenine and 3-indoxylsulfate (synthesized in the liver) were noted to be higher in the ALAC ILA non-producers, whereas ILA (produced in the distal gut by gut microbiota) was only higher in ALAC ILA producers, we hypothesize that there is an organ-specific response in the AhR-dependent signaling pathway due to the difference in AhR agonists within those piglets fed the same diet.

### Gut microbial activity mediated dietary effect on gene expression profiles

**Fig. 5:**
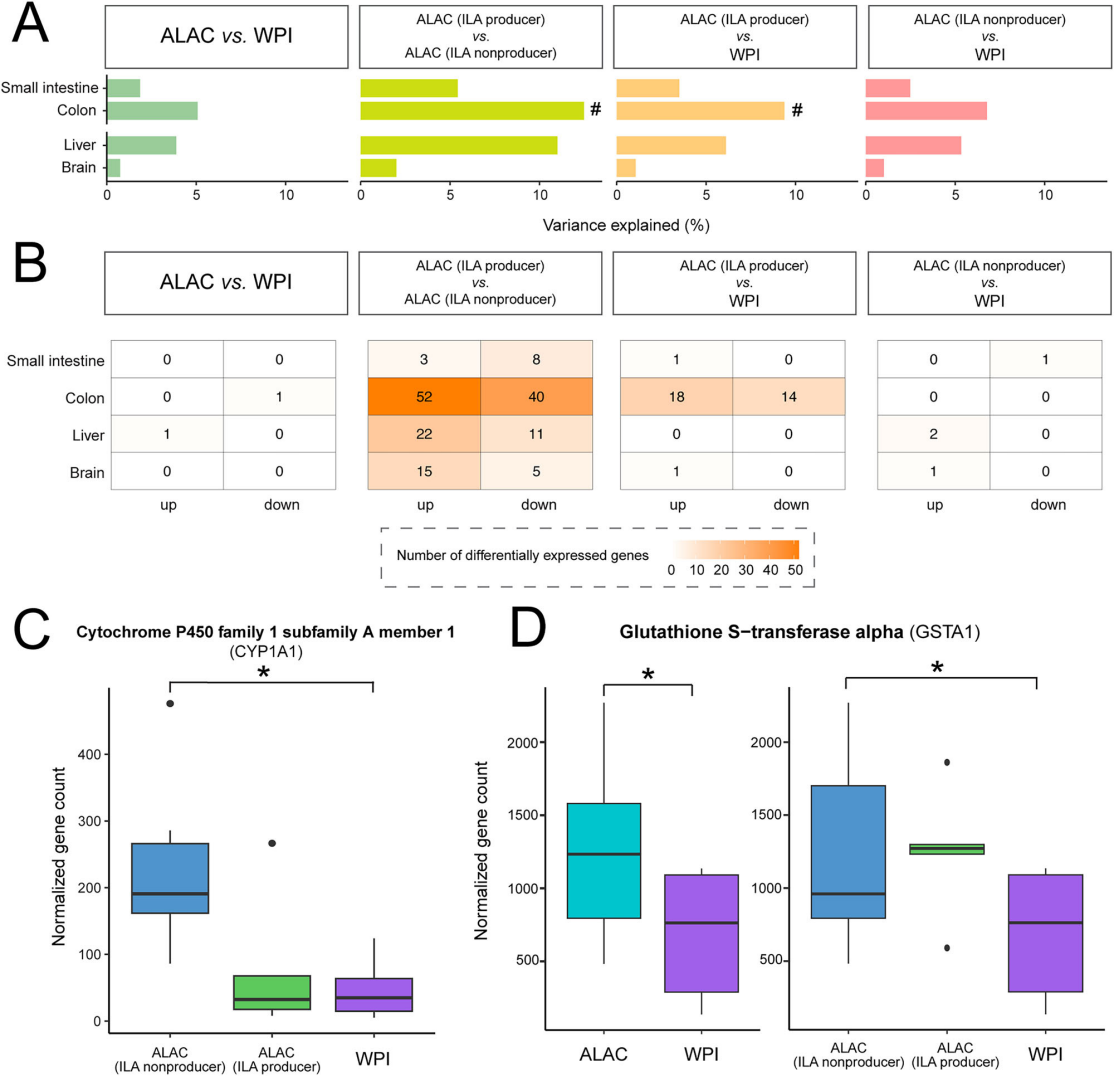
Transcriptomics reveals the divergent effects of diet on the intestine, brain, and liver. **A** Permutational multivariate analysis of variance (PERMANOVA) using the adonis function with Canberra distance on RNA-seq data. The Benjamini-Hochberg FDR procedure was applied to adjust for multiple hypothesis testing. No adjusted *p*-values fell below 0.05. Instances where unadjusted *p*-values are less than 0.05 are marked with a hash (#). **B** The number of up- and downregulated genes between groups. The group difference was evaluated using DeSeq2, followed by Benjamini & Hochberg FDR correction with a minimum 1.5-fold change difference. The complete list of differentiating genes is in Supplementary Table 3. **C**, **D** Gene expression of cytochrome P450 family 1 subfamily A member 1 (CYP1A1) and glutathione s-transferase alpha (GSTA1) in the liver. * Adjusted *p* < 0.05.

Transcriptome analysis provides an advanced approach to elucidating possible molecular mechanisms responsible for variability in response to diet. To further understand the diet-microbe-host interaction, genome-wide RNA-seq was performed on the ileum, jejunum, colon, liver, hippocampus, hypothalamus, striatum, and prefrontal cortex collected on Day 16. The small intestine, colon, liver and brain transcriptomics profiles of piglets fed two different formulas were subtle; however, differences between the ALAC ILA producer and ILA non-producer, as well as the ALAC ILA producer and the WPI group were more profound in the colon (Fig. 5A). Here, more differentially expressed genes were observed that could be associated with the microbial ILA production phenotype (Fig. 5B).

Functional gene ontology analysis further linked some of these ILA-associated up-regulated genes with defense responses to viruses and symbionts (biological process, GO: 0051607, 0140546) (Table 1). Interestingly, immune response genes, including radical S-adenosyl methionine domain containing 2 (RSAD2), interferon-induced protein with tetratricopeptide repeats 1 (IFIT1), and interferon-induced protein with tetratricopeptide repeats 3 (IFIT3) were found up-regulated in the colon, liver, and brain of ILA producers, suggesting a remote communication between the gut and distal organs such as the liver and the brain with respect to host immune defense.

**Table 1:** Differentially expressed genes in the colon, liver, and brain that are associated with defense response to viruses and symbionts

Gene name	Colon				Liver		Brain	
	ALAC (ILA producer) vs ALAC (ILA non-producer)		ALAC (ILA producer) vs. WPI		ALAC (ILA producer) vs ALAC (ILA non-producer)		ALAC (ILA producer) vs ALAC (ILA non-producer)	
	Log2 Fold Change	Adj. *p*	Log2 Fold Change	Adj. *p*	Log2 Fold Change	Adj. *p*	Log2 Fold Change	Adj. *p*
Radical S-adenosyl methionine domain containing 2 (RSAD2)	1.958	0.001	0.927	0.033	1.579	<0.001	2.516	<0.001
Interferon-induced protein with tetratricopeptide repeats 1 (IFIT1)	1.908	0.002	1.331	0.033	1.159	<0.001	0.873	<0.001
Interferon-induced protein with tetratricopeptide repeats 3 (IFIT3)	1.865	0.011	1.805	<0.001	1.061	0.007	0.818	<0.001
Interferon-induced protein with tetratricopeptide repeats 2 (IFIT2)	1.614	0.028	1.55	0.014			0.836	0.0126
RNA sensor RIGI (RIGI)	1.475	<0.001	0.99	0.006	1.19	<0.001		
Myxovirus (influenza virus) resistance 2 (mouse) (MX2)	1.322	0.015	0.899	0.021			1.202	<0.001
Interferon induced with helicase C domain 1 (IFIH1)	1.024	<0.001	0.609	0.012			0.684	<0.001
Poly(ADP-ribose) polymerase family member 9 (PARP9)	0.822	0.004						
Zinc finger NFX1-type containing 1 (ZNFX1)	0.762	0.018			0.843	0.004		
HECT and RLD domain containing E3 ubiquitin protein ligase 5 (HERC5)	0.752	0.02			0.995	0.018		
Signal transducer and activator of transcription 1 (STAT1)	0.631	0.014			0.667	0.005		
Eukaryotic translation initiation factor 2 alpha kinase 2 (EIF2AK2)	0.587	0.015						
Z-DNA binding protein 1 (ZBP1)	1.181	0.002						
A-kinase anchoring protein 1 (AKAP1)			0.638	0.043				
Interferon-induced protein 44-like (IFI44L)			0.593	0.011			0.869	<0.001
MX dynamin-like GTPase 1 (MX1)					0.846	0.014		
DExD/H-box helicase 60 (DDX60)					0.876	0.01		
2′-5′-Oligoadenylate synthetase 2 (OAS2)					1.063	0.007		
Deltex E3 ubiquitin ligase 3L (DTX3L)							0.782	<0.001
C-X-C motif chemokine ligand 10 (CXCL10)							1.085	<0.001

In the liver, the cytochrome P450 family 1 subfamily A member 1 (CYP1A1) and glutathione S-transferase alpha (GSTA1), both target genes that are induced by AhR signaling, were found significantly up-regulated in the ALAC ILA non-producers compared to the WPI group (Fig. 5C, D). No AhR-regulated genes were differentially expressed in the liver, small intestine, and colon of the ALAC ILA producer group compared to the WPI group. Collectively, these findings illustrate inter-individual variability in the divergent metabolic impacts of diet due to differences in host digestion and absorption efficiency of tryptophan, as well as gut microbial production of ILA.

## Discussion

In this study, we attempted to untangle some aspects of the complex diet-microbiota-host crosstalk. We provide evidence from a neonatal piglet model to support that altering whey protein source in the formula itself is insufficient to manipulate gut microbial fermentation patterns in the GIT. However, consumption of a diet enriched with α-lactalbumin (a rich dietary source of tryptophan) can trigger a personalized response that is associated with individual variabilities in host and gut microbial tryptophan metabolism.

The early gut microbiota undergoes rapid changes soon after birth, transitioning toward an adult-like configuration. As demonstrated in both human infants and piglets, microbial diversity progressively increases after birth, exhibiting high intra- and inter-individual variability before reaching a more stable composition (piglet: after weaning; human infants: at the age of approximately 3 years)^22–24^. The instability due to the fast-changing maturation process may be a key contributor to individual variability. Furthermore, the GIT is the body’s largest immune organ. Host immune-intestinal microbiota interaction during this critical window of opportunity in early life could have a long-lasting impact and contribute to immune homeostasis and susceptibility to inflammatory diseases and metabolic disturbances later in life^25–27^.

Although the majority of milk proteins are considered to be highly digestible and absorbable by infants to provide amino acids and energy, some proteins are subject to partial proteolysis, exhibiting a wide range of biological actions, including anti-pathogenic activity and immunomodulating properties, all of which promote the healthy development of infants^28,29^. α-Lactalbumin is one of those functional proteins in milk^30,31^. Although no intact α-lactalbumin has been detected in the stool of infants fed breastmilk, digestion of α-lactalbumin is relatively slow, remaining intact for at least 1 h in the stomach post-feeding^30,32^. Here, we show that in some of these piglets fed a formula with an α-lactalbumin-enriched whey protein isolate, a small portion of the ingested tryptophan in the colon and cecum serves as a substrate that stimulates the production of ILA by intestinal microbes. Furthermore, the degree of protein digestion and absorption in the small intestine results in variation in microbial protein catabolism in the distal GIT, and at the same time influences tryptophan availability for host utilization.

Tryptophan in infant formula, if efficiently absorbed in the small intestine after a meal, may exhibit various biological activities beyond providing building blocks of proteins and a source of energy. For example, tryptophan-derived metabolites by the host liver, including kynurenine and 3-indoxylsulfate, are endogenous agonists of AhR, which has a significant role in immune regulation and protection against pathogen infection^21^. Furthermore, gene expression of xenobiotic-metabolizing enzymes from the cytochrome P450 superfamily is regulated by an AhR-dependent control, which plays a protective role in cellular detoxification of a wide variety of xenobiotics^21,33^. The release of reactive oxygen species (ROS) that is induced by excessive AhR-mediated activity can be counteracted by AhR-mediated antioxidative activities from glutathione s-transferase and NAD(P)H quinone dehydrogenase 1 (NQO1)^33^. In this study, we confirm the upregulation of two AhR-targeted xenobiotic-responsive genes (CYP1A1 and GSTA1) in the liver of piglets that had more efficient host utilization of dietary tryptophan. We speculate that elevated kynurenine and 3-indoxylsulfate may be a diet-induced target that can efficiently regulate the expression of AhR-targeted xenobiotic-responsive genes in a tissue-specific manner. Although these findings are crucial for understanding the underlying effect of this complex diet-host interaction, whether these observed effects translate into immediate or long-term phenotypic impacts remains to be elucidated.

In contrast, if more tryptophan reaches the distal GIT after a meal, it can directly interact with microbes that have tryptophan utilization capability to produce microbially-derived tryptophan metabolites^34,35^. ILA, a microbial catabolite of tryptophan, has been identified as an anti-inflammatory metabolite that is enriched in the stool of human breastfed infants with a *Bifidobacterium*-dominant intestinal microbiome^36,37^. Previously, ILA has been reported in vitro to have direct anti-fungal activity against *Penicillium* spp.^38^ and antibacterial activity against foodborne pathogens such as *Salmonella* spp., *Staphylococcus* spp., *Escherichia coli*, *Listeria monocytogenes*, and *Bacillus cereus*^38,39^. One host immune regulatory action of ILA is through its interaction as a ligand with the AhR-dependent pathway^37,40,41^. In addition, ILA can also promote intestinal immune homeostasis and cell development via the signal transducer and activator of transcription 1 (STAT1) pathway in immature enterocytes^42^. Lastly, ILA has shown viral inhibitory potential by up-regulating gene expression of anti-viral genes such as IFIT2 and RSAD2^42^. Although ILA is notable for its immune modulation capability, its potential effects on the systemic level remain to be elucidated.

Our results are consistent with a previous report on rodents and cell culture^42^, but add more immunological details. Under an experimental condition that mimics normal human infant feeding practice, we demonstrated upregulation of STAT1 and viral and symbiont defense genes (RSAD2, IFIT1, IFIT3, and RIGI) in healthy piglets with detectable ILA in the distal GIT; however, a direct gene expression level change in the AhR-signaling pathway was not observed. Under normal physiological conditions, the ILA-induced upregulation of viral and symbiont defense genes could serve as an underlying mechanism that supports the protective role of human milk in reducing the frequency and duration of infection.

Although tryptophan is the necessary substrate to induce functional microbial by-products, ingesting a high level of free tryptophan for the purpose of promoting microbial fermentation is not feasible, as most tryptophan is readily absorbed in the small intestine and can quickly shunt to tryptophan catabolism in the liver via the kynurenine pathway. Simply aiming for excessive free tryptophan levels in the diet does not guarantee its availability for intestinal microbes, and can be harmful by adversely affecting the morphology of the intestinal epithelium and tight junction proteins^43^. In comparison to pure tryptophan, a tryptophan-rich protein source such as α-lactalbumin allows gastric and intestinal proteolysis to take place. Approximately 10% of bovine α-lactalbumin is glycosylated^44^, which may increase the protein’s resistance to digestion.

This study demonstrates that inter-individual variation is linked to different responses of the same diet on host phenotypes, which is independent of genetics. The use of a neonatal piglet model allowed for careful accounting of variations in diet, formula intake, and feeding time, and allowed us to evaluate functional markers in digestive content and key metabolic tissues, all of which are essential for mechanistically assessing the complex interrelationship between diet, host, and gut microbiota. Stratifying individuals into subgroups based on their microbial functional attributes defined by quantitative metabolomics analysis provides a promising approach to identifying individual-specific responses to a given diet. Our results suggest that unique host nutrient utilization efficiencies and diet-induced microbial signatures could be sources that contribute to individual variation, and the role of gut microbiota on dietary response appears more complex than previously thought. A one-size-fits-all diet approach is unlikely to elicit a consistent effect across all individuals. Therefore, it is important to highlight the need for further investigation into the role of microbial-diet interaction and its impact on early development.

## Methods

### Animal study

A total of 30 piglets were obtained from 3 sows (Hampshire × Yorkshire × Landrace cross). To ensure proper immune development, all piglets were allowed to stay with their respective sow from birth to Day 5. On Day 3, all piglets received 200 mg of elemental iron as iron dextran, and male piglets were castrated. Starting from Day 6 until Day 16, piglets were weight-, sex- and litter- matched, and randomly assigned into 1 of 3 dietary groups: ALAC (*n* = 12, 6 males and 6 females), who received a formula enriched in α-lactalbumin (Lacprodan® ALPHA-50, Arla Foods Ingredients Group P/S, Denmark), WPI (*n* = 12, 6 males and 6 females), who received a formula containing a standard whey protein isolate (Lacprodan® DI-9224, Arla Foods Ingredients Group P/S, Denmark) that is primarily β-lactoglobulin, and piglets that remained with their birth sow (sow-fed (SF)) (*n* = 6, 3 males and 3 females). On Day 7, 1 SF piglet was euthanized due to sow trauma, and 1 piglet that was initially randomized to the ALAC group repeatedly jumped the barrier to return to her sow and was allowed to remain. As a result, a total of 11 ALAC (6 males, 5 females), 12 WPI piglets (6 males, 6 females), and 6 SF piglets (3 males, 3 females) completed the study. The schematic of the study is illustrated in Fig. 1B. Body weight was recorded daily to assess growth and determine daily formula requirement.

Piglets are social animals and are playful throughout their development. To ensure normal behavioral, social, and cognitive development, ALAC and WPI piglets were group-housed in two farrowing pens during the intervention. Although all formula-fed piglets quickly learned to suckle from a nursing bottle, slow weight gain was observed during the first two days of formula introduction, despite sufficient volume of formula being provided to match their daily caloric requirement. On Day 11 or 12, ALAC and WPI piglets underwent postprandial blood collection, which resulted in a lower formula intake and a subsequent decline in weight gain. By the end of the intervention, ALAC and WPI piglets were unable to catch up in growth and were significantly smaller than the sow-fed counterparts, but still within normal range for formula-fed piglets. This result was reported previously^45^.

### Diet design and preparation

Sow milk and human milk are compositionally different; therefore, human infant formula cannot be directly used on neonatal piglets. To establish a reference for piglet formula, we first analyzed milk from a lactating sow pooled from postpartum day 6 and 14. The nutrient composition and the major ingredients used in diet formulation are provided in Supplementary Tables 1 and 2. The two piglet formulas were produced at the Milk Processing Laboratory at UC Davis. The details of piglet formula production are available in Shoff et al.^45^.

### Sample collection

On Day 11 or 12, ALAC and WPI piglets were subjected to a blood draw from the cephalic vein after fasting for at least 5 h. After the baseline blood collection, ALAC and WPI piglets were subsequently allowed to consume up to 120 mL of the assigned formula for a maximum of 15 min. After the meal, piglets were returned to their respective pen, and blood samples were drawn at 30-, 60-, and 120- min post-meal.

On Day 16, prior to sacrifice, the ALAC and WPI piglets were fasted for approximately 5 h, then allowed to consume up to 120 mL of formula for up to 15 min, and were sacrificed at approximately 1 h post-meal. To achieve a similar postprandial time in the SF piglets, sows nursing the SF piglets were injected with 20 USP units of oxytocin 90 min prior to sacrifice to encourage milk production and let down approximately 1 h prior to sacrifice. Piglets from all three groups were anesthetized using a premixed solution combining Telazol™, ketamine, and xylazine, followed by cardiac puncture and euthanized through intracardiac injection of pentobarbital (FatalPlus™, Vortech Pharmaceuticals, Dearborn, MI).

Blood drawn from the cephalic vein on Day 11 or 12, or from cardiac puncture on Day 16, was collected into serum separation tubes. Serum was separated by centrifugation at 1600 × *g* for 15 min at 4 °C. Aliquots of serum samples were stored at −80 °C until further analysis. Immediately after euthanasia, brain regions (hypothalamus, hippocampus, striatum, prefrontal cortex), liver (the left lateral lobe), intestinal tissues (ileum, jejunum, colon), urine, and digestive content of the stomach, duodenum, jejunum, ileum, cecum, and colon were collected, followed by snap-freezing in liquid nitrogen and storage at −80 °C until further analysis.

### Targeted NMR-based metabolomics of serum, urine, liver, brain and intestinal content

Measurements of tyrosine, histidine, isoleucine, valine, threonine, niacinamide in serum from Day 16, measurements of tyrosine, histidine, isoleucine, valine and NAD^+^ in liver, measurements of tyrosine, isoleucine, valine, and threonine in brain regions, measurements of tryptophan, quinolinate, niacinamide, 1-methylnicotinamide, N-methyl-2-pyridone-5-carboxamide, 3-indoxylsulfate in urine were determined via ^1^H-NMR-based targeted metabolomics.

A total of 82 metabolites were quantified in the digestive contents of stomach, duodenum, jejunum, ileum, cecum and colon, which included 1,3-dihydroxyacetone, 2-hydroxyglutarate, 2-hydroxyisobutyrate, 2-hydroxyisocaproate, 2-hydroxyisovalerate, 2-oxoglutarate, 3-phenyllactate, 4-aminobutyrate, 4-hydroxyphenylacetate, 4-hydroxyphenyllactate, 5-aminopentanoate, acetate, alanine, arginine, asparagine, aspartate, betaine, bile acids, butyrate, cadaverine, choline, citrate, creatine, creatinine, cystine, cytosine, desaminotyrosine, dimethylamine, ethanolamine, formate, fumarate, galactose, glucose, glutamate, glutamine, glycerol, glycerophosphocholine, glycine, histamine, histidine, hypoxanthine, indole-3-acetate, indole-3-lactate, inosine, isobutyrate, isoleucine, isovalerate, lactate, lactose, leucine, lysine, malate, methanol, methionine, methionine-sulfoxide, methylamine, nicotinate, O-phosphocholine, ornithine, phenylacetate, phenylalanine, proline, propionate, putrescine, pyruvate, ribose, serine, succinate, taurine, threonine, thymine, trimethylamine, tryptophan, tyramine, tyrosine, UMP, uracil, urea, valerate, valine, *myo*-inositol, and *p*-cresol.

#### Serum and urine

Serum and urine samples were filtered through Amicon Ultra-0.5 mL centrifugal filters with a cutoff of 3000 MW (Millipore, Billerica, MA). To 207 μL of filtrate, 23 μL of internal standard containing 4.608 mM 3-(trimethylsilyl)-1-propanesulfonic acid-d6 (DSS-d6), 0.2% NaN_3_, and 99.8% D_2_O was added. Each sample was then adjusted to a pH of 6.85 ± 0.1.

#### Liver and brain

Approximately 75 mg of liver tissue was extracted using a modified Folch extraction described by Hasegawa et al.^46^. The polar metabolites were reconstituted in phosphate buffer made with D_2_O. To 207 μL of supernatant, 23 μL of internal standard containing 4.608 mM 3-(trimethylsilyl)-1-propanesulfonic acid-d6 (DSS-d6), 0.2% NaN_3_, and 99.8% D_2_O was added. Each sample was then adjusted to a pH of 6.85 ± 0.1 through the addition of small amounts of NaOH or HCl.

#### Gastrointestinal contents

Approximately 200 mg of digestive content from the stomach, duodenum, jejunum, ileum, cecum, and colon were extracted by mixing with 1 mL of ice-cold phosphate-buffered saline (40 mM). To remove any excess proteins and lipids, the supernatant was filtered through a 0.22 μm filter, followed by a 3000 MW cutoff ultra-centrifugal filter (Amicon, Millipore, MA, USA). 207 μL of the filtrate was combined with 23 μL of an internal standard 5 mM DSS-d6 (trimethylsilyl-propane-sulfonate) containing 0.2% NaN_3_ in 99.8% D_2_O to allow for quantification of metabolites. The pH of each sample was adjusted to 6.8 ± 0.1 through the addition of small amounts of NaOH or HCl.

#### NMR data acquisition and spectral analysis

All ^1^H-NMR spectra were collected at 25 °C using the noesypr1d pulse sequence on a Bruker Avance 600 MHz spectrometer (Bruker, Billerica, MA) as described^47^. Chenomx NMR Suite (version 8.6, Chenomx, Edmonton, AB) was used to process and analyze the NMR spectra. Each spectrum was Fourier transformed, and then manually phase and baseline corrected in Processor. Metabolites were quantified by the same researcher in Profiler, using the concentration of the internal standard (DSS-d6) as reference.

All metabolites (except bile acids) measured from serum and gastrointestinal content are expressed in μmol/L (μM). Total bile acid in the gastrointestinal tract was determined via spectral binning targeting 0.65–0.75 ppm with internal reference area normalization and dilution correction. The concentration of brain and hepatic metabolites are expressed in nmol/g. The concentration of urine metabolites was normalized by effective osmolality and expressed in µM/Osm. Urine osmolality was first measured in duplicate using a VAPRO vapor pressure osmometer (ELITech, Logan, UT). Although urinary urea contributes to measured urine osmolality, since it freely crosses cell membranes, therefore, it does not contribute to tonicity. The effective osmolality is determined as measured osmolality minus the molar contribution of the urinary urea to osmolarity.

### Untargeted and Targeted GC-MS-based metabolomics of serum, liver and brain

Measurements of serum tyrosine, histidine, isoleucine, valine, and threonine on Day 11 or 12 were assessed using untargeted GS-MS metabolomics, and serum, liver, and brain concentrations of tryptophan, quinolinate, kynurenine, and serotonin were quantified using targeted GS-MS metabolomics. Untargeted and targeted data were generated by the West Coast Metabolomics Center (University of California Davis, Davis, CA) as previously described^48^. In short, samples were extracted in 1 mL of 3:3:2 CAN:IPA:H_2_O (v/v/v). Samples were split into two equal aliquots. One aliquot was completely dried and derivatized using 10 µL of 40 mg/mL of methoxyamine in pyridine, and shaken for 90 min at 30 °C. In addition to the incorporation of a mixture of internal markers (described previously^48^) for quantitative purposes, 91 µL of N-Methyl-N-(trimethylsilyl)trifluoroacetamide (MSTFA) was added, followed by additional shaking for 30 min at 37 °C to complete derivatization. Data were collected using a 7890 Agilent GC (Agilent Technologies, Santa Clara, CA) coupled to a Leco Pegasus IV TOFMS (Leco, St. Joseph, MI). A total of 0.5 µL of derivatized sample was injected using a splitless method onto an RTX-5SIL MS column with an Intergra-Guard (Restek, Centre County, PA) at 275 °C with a helium flow of 1 mL/min. Oven temperature was set to hold at 50 °C for 1 min, ramp to 300 °C at 20 °C/min, then hold for 5 min. Ion source temperature was 250 °C, and mass spectral acquisitions were collected from 80 m/z to 500 m/z at 17 spectra/s. For targeted data, a curve was injected from 0.1 to 20 µg/mL, which was acquired alongside the samples. The concentrations of serum metabolites are expressed in μmol/L (μM). The concentration of liver and brain metabolites are expressed in nmol/g.

### RNA-seq analysis of liver, brain, and intestinal tissue

#### RNA isolation

RNA was extracted as described in Hasegawa et al.^46^. In short, tissues were manually ground in liquid nitrogen using a mortar and pestle. 25–30 mg of cryoground tissue was extracted using a modified Folch extraction, and RNA was extracted from the resulting cell layer (All Prep DNA/RNA/Protein Kit, Qiagen, Germantown, MD). RNA samples were quantified using the Qubit RNA HS Assay Kit and Quibit 3.0 Fluorometer (Thermo Fisher Scientific, Waltham, MA), and sample quality was assessed using a Lab Chip GX Touch HT (PerkinElmer, Waltham, MA).

#### Library preparation and sequencing

To generate Illumina-compatible multiplexed sequencing libraries, barcoded 3′Tag-Seq libraries were prepared using the QuantSeq FWD kit (Lexogen, Vienna, Austria) according to the manufacturer’s recommendations. The fragment size distribution of the libraries was verified via micro-capillary gel electrophoresis via Bioanalyzer 2100 (Agilent, Santa Clara, CA). The libraries were quantified fluorometrically on a Qubit instrument (LifeTechnologies, Carlsbad, CA), and pooled in equimolar ratios. 232 libraries were sequenced on 2.5 lanes of NextSeq500 sequencer (Illumina, San Diego, CA), generating on average 3.7 million forward reads per sample, with a sequence length of 85 bp.

#### RNA-seq analysis

The RNA-seq reads quality was evaluated via FastQC (v0.14.0)^49^, FastQ Screen (v0.14.0)^50^, and QualiMap (v2.1.1)^51^. Removal of low-quality reads/nucleotides and adapter sequences was performed via BBDuk from the BBTools suite (v38.72) using the recommended parameters^52^ for the QuantSeq experiment. The trimmed-high-quality sequencing reads were then mapped to the latest reference genome for domestic pig (Sscrofa11.1)^16^ using STAR aligner (v2.7.10a)^53^ with the recommended modified-Encode settings. Finally, quantification of per-gene counts was obtained using HT-Seq count (v2.0.2)^54^. Count-based differential expression analysis was performed using DESeq2^55^. A list of differentially expressed genes was selected with a fold change >1.5 and *p* < 0.05 by the default Wald test with Bonferroni-Hochberg multiple testing adjustment. Gene ID conversion tool from DAVID Bioinformatics Resources^56^ was used to match an Ensembl gene ID to a gene name.

### SDS-PAGE gel electrophoresis of the intestinal content

To determine the intestinal content dry weight, an aliquot of intestinal content was lyophilized (Labconco FreeZone 4.5 L Freeze Dry System, Labconco, MO). Protein was extracted from intestinal contents according to the method described by Hasegawa et al.^46^. The protocol was modified to be appropriate for wet samples by manually homogenizing intestinal contents or formula, then extracting a wet weight equivalent to 25 mg of dry weight. The resulting protein pellet was reconstituted in 5% SDS, and protein was quantified using the DC Protein Assay Kit II (Bio-Rad, Hercules, CA) using a Synergy H1 Plate Reader (BioTek, Winooski, VT). 30 µg of protein separated on TGX Stain-Free gels (AnyKD™, Bio-Rad, Hercules, CA) alongside a ladder (Precision Plus™ Unstained Protein Ladder, Bio-Rad, Hercules, CA), which was then imaged (ChemiDoc MP, Bio-Rad, Hercules, CA). 30 µg of ALAC powder used in formula development, dissolved in 5% SDS, was used for comparative purposes.

### Statistical analysis

Statistical computing and graphical generation were performed using the R programming environment. Plots were generated using *ggplot* or *ggpubr*. In brief, generalized log transformation (defined as *log(y+sqrt(y^2 + lambda)*) was applied to all metabolomics data where *lambda* is 1. Group differences were evaluated using ANOVA followed by Benjamini & Hochberg FDR multiple comparison correction on generalized log-transformed data. The effect size was determined via generalized eta squared using *rstatix*. Confounding factors (litter, multiple time points, regions, formula volume, time since meal) were accounted for in the model where necessary. Pearson’s correlation coefficient (*r*) was used to evaluate the strength of correlation. Non-metric multidimensional scaling (NMDS), permutational multivariate analysis of variance (PERMANOVA), and Mantel tests were performed on Canberra distances using vegdist, *metaMDS(k* = *2, trymax* = *30), adonis(by* = *“margin”, nperm* = *999),* and *mantel* from the *vegan* package. The overall level of significance was set at *p* < 0.05.

### Ethical approval

Animal handling was approved by the University of California Davis (UC Davis) Institutional Animal Care and Use Committee (IACUC) and registered under protocol #22011. All experiments were performed in accordance with relevant guidelines and regulations, adhering to the UC Davis Swine Teaching and Research Center husbandry protocols.

## Supplementary information

He et al. Supplementary data

## Data Availability

All data and scripts used for analysis and figure generation are made available at xuahe.github.io/piglet-ila-variability-study. The RNA-seq data can be found at GEO Accession GSE281726.

## Code availability

All data and scripts used for analysis and figure generation are made available at xuahe.github.io/piglet-ila-variability-study. The RNA-seq data can be found at GEO Accession GSE281726.
