# Supplementary material for: Functional gut variability drives metabolic and transcriptional features in neonatal piglets
Source: NPJ Sci Food. 2025 Oct 16;9:207. doi: 10.1038/s41538-025-00571-z (PMC12533098; doi:10.1038/s41538-025-00571-z)
Supplement: Supplementary file 1 — He et al. Supplementary data [file 41538_2025_571_MOESM1_ESM.pdf]

Supplementary information

Functional gut variability drives metabolic and transcriptional features in neonatal piglets

Xuan He, Shannon Shoff, Hanna Lee, Merete Lindberg Hartvigsen, Anne Staudt Kvistgaard, Carolyn M Slupsky

Table of Contents

1. **Supplementary Figures**

- **Supplementary Fig. 1.** Serum tryptophan concentration at Day 16.
- **Supplementary Fig. 2.** SDS-PAGE gel of intestinal content at Day 16.

2. **Supplementary Tables**

- **Supplementary Table 1.** Nutrient composition of the ALAC diet, the WPI diet and sow milk.
- **Supplementary Table 2.** Major source of ingredients used in the diet formulation.
- **Supplementary Table 3.** Differentially expressed genes in small intestine, colon, liver and brain.

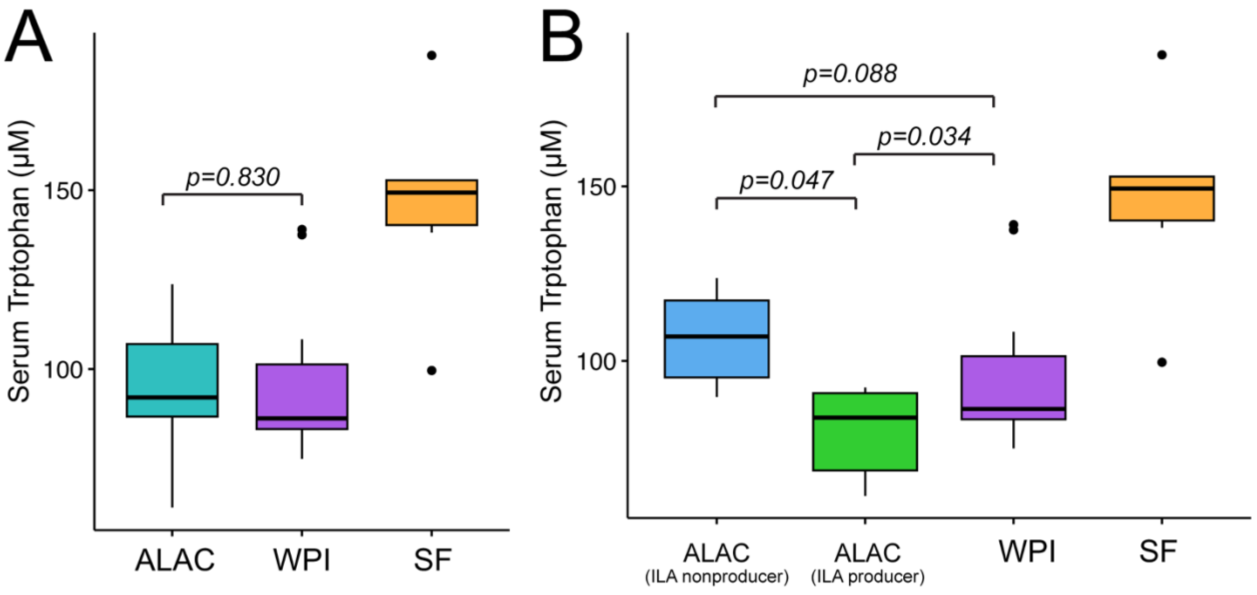

Supplementary Fig. 1. Serum tryptophan concentration at Day 16.

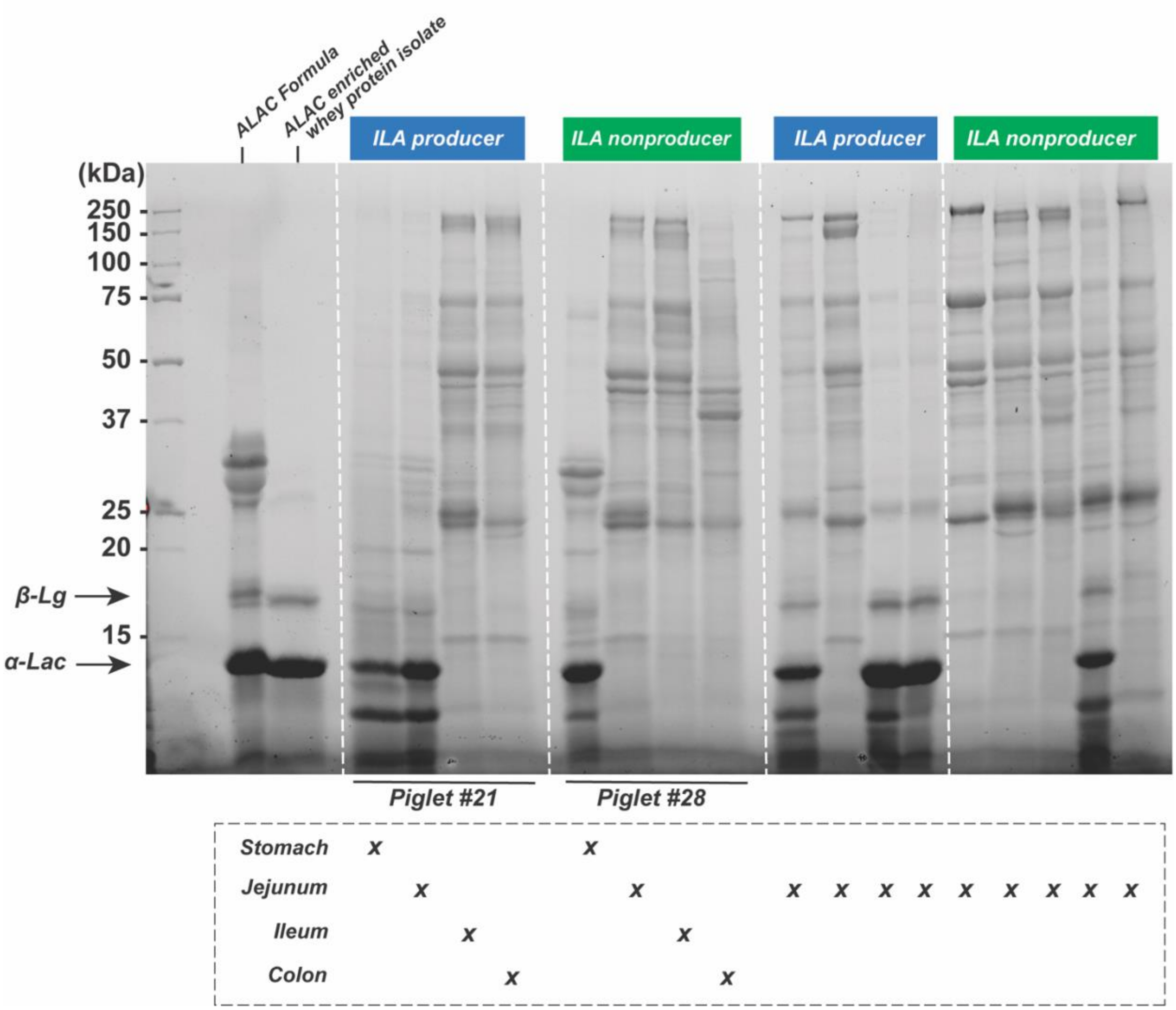

Abbreviations: α-Lac, α-Lactalbumin; β-Lg, β-Lactoglobulin.

Supplementary Fig. 2. SDS-PAGE gel of intestinal content at Day 16. Band intensity corresponds to α-lactalbumin at approximately 14 kDa was used to generate visualizations at Fig. 3B,D.

Supplementary Table 1. Nutrient composition of the ALAC diet, the WPI diet and sow milk.

	ALAC diet	WPI diet	Sow milk	
	Formula made with α-lactalbumin enriched whey protein isolate	*formula made with standard whey protein isolate*	*Quantified using pooled sow milk*	*Literature values*
Energy (kcal/L)	1068.2	1063.2		1140[1]
Carbohydrate *(g/L)*	56.55	56.55		
Lactose (g/L)	*55.00*	*55.00*	*54.5*	*54[1]; 41.1-55.3[2]*
Corn starch (g/L)	*1.55*	*1.55*		
Protein (g/L)	44.30 ± 0.85	43.15 ± 0.78	46.19	42.2[1]; 48.3-50.4[2]; 54[3]
Whey:casein ratio	*49.5:50.5*	*50:50*		*50:50[4]*
α-Lactalbumin (g/L)	*18.33*	*1.83*		
β-Lactoglobulin (g/L)	*1.43*	*12.94*		
Casein glycomacropeptide (g/L)	*0.00*	*4.25*		
Fat (g/L)	70.00	70.00		71[1]; 73.3-78.4[2] ; 76[3]
Amino acid composition (g/L)				
Alanine	1.00 ± 0.00	1.66 ± 0.03	1.52	2.47[1]
Arginine	1.65 ± 0.04	1.68 ± 0.01	2.13	0.89[1]
Arg from protein	*0.72 ± 0.04*	*0.97 ± 0.01*		
Addition of free Arg	*0.93*	*0.70*		
Aspartate/asparagine	4.89 ± 0.08	3.62 ± 0.05	3.86	4.21[1]
Methionine	1.00 ± 0.04	1.12 ± 0.00	1.07	0.74[1]
Met from protein	*0.81 ± 0.04*	*1.12 ± 0.00*		
Addition of free Met	*0.20*	*0.00*		
Cysteine	1.16 ± 0.05	0.62 ± 0.01	0.68	0.65[1]
Glutamate/glutamine	7.80 ± 0.23	8.01 ± 0.07	9.21	6.83[1]
Glu/Gln from protein	*6.85 ± 0.23*	*8.01 ± 0.07*		
Addition of free Glu	*0.95*	*0.00*		
Glycine	0.89 ± 0.01	0.88	1.18	1.64[1]
Gly from protein	*0.78 ± 0.01*	*0.43*		
Addition of free Gly	*0.11*	*0.45*		
Histidine	1.09 ± 0.00	0.97	1.32	0.65[1]
His from protein	*1.09 ± 0.00*	*0.77*		
Addition of free His	*0.00*	*0.20*		
Isoleucine	2.24 ± 0.04	2.39 ± 0.08	1.96	1.94[1]
Leucine	4.07 ± 0.08	4.11 ± 0.08	3.79	3.71[1]
Lysine	3.89 ± 0.03	3.62 ± 0.04	3.60	3.10[1]
Phenylalanine	1.89 ± 0.06	1.59 ± 0.01	1.81	1.40[1]
Tyrosine	1.98 ± 0.05	1.64 ± 0.00	1.95	1.23[1]
Proline	3.70 ± 0.06	3.74 ± 0.04	4.71	5.33[1]
Pro from protein	*1.99 ± 0.06*	*2.97 ± 0.04*		
Addition of free Pro	*1.71*	*0.77*		
Serine	1.97 ± 0.05	2.03 ± 0.01	2.36	2.56[1]
Threonine	1.95 ± 0.01	2.35 ± 0.02	2.08	2.15[1]
Tryptophan	1.03 ± 0.05	0.89 ± 0.10	0.66	0.35[1]
Valine	2.12 ± 0.04	2.44 ± 0.03	2.30	2.39[1]
Taurine	166.00	166.00	165.9	

Nutrient composition of each piglet formula was based on amounts and purity of ingredients used in production, as well as results from total amino acid analysis. After diet production, total amino acids (except glycine and histidine from the WPI formula) were measured from two production batches. The concentration of glycine and histidine from the WPI formula were determined from a single replicate. Amino acid results are expressed in mean ± standard deviation. Concentration of lactose, *α-*

lactalbumin, *β*-lactoglobulin, casein glycomacropeptide, and free amino acids in the pig formulas were estimated based on ingredient product information and added quantity.

Total amino acids in sow milk were measured using a pooled milk sample containing 39.5% of the volume from postpartum day 6, 5.4% from postpartum day 11, and 55.1% from postpartum day 14. The total protein concentration of measured sow milk was as determined as the sum of total amino acids. Lactose in sow milk was quantified using ^{1}H NMR and determined as the average of measurements from postpartum day 6 and 14.

Supplementary Table 2. Major source of ingredients used in the diet formulation

**Nutrients**	**ALAC diet**<br>*Formula made with α-lactalbumin enriched whey protein isolate*	**WPI diet**<br>*Formula made with standard whey protein isolate*
Lactose	Lactose, 200 Mesh, Leprino Foods Dairy Products Company, CO, USA	
Whey	Alpha-50, Arla Foods Ingredients, Denmark	Lacprodan DI-9224, Arla Foods Ingredients, Denmark
Casein	Micellar Casein, Leprino Foods Dairy Products Company, CO, USA	
Fat	Soybean oil 100, Envigo, IL, USA; Kirkland signature soybean oil, TN, USA	
Vitamins	Custom vitamin premix, Envigo, IL, USA	
Minerals	In-house mineral premix	

Vitamins include *p*-aminobenzoic acid, coated vitamin C, biotin, vitamin B_{12}, calcium pantothenate, choline dihydrogen citrate, folic acid, inositol, vitamin K_3, niacin, pyridoxine HCl, riboflavin, thiamin, vitamin A palmitate, vitamin D_3 cholecalciferol, vitamin E DL-α-tocopheryl acetate.

Minerals include calcium phosphate monobasic monohydrate, calcium acetate monohydrate, potassium citrate monohydrate, potassium chloride, magnesium acetate tetrahydrate, zinc sulfate heptahydrate, manganese sulfate tetrahydrate, copper sulfate, potassium iodate and sodium selenite

References

1. Odle, J., Lin, X., Jacobi, S. K., Kim, S. W. & Stahl, C. H. The Suckling Piglet as an Agrimedical Model for the Study of Pediatric Nutrition and Metabolism. *Annu. Rev. Anim. Biosci.* **2**, 419–444 (2014).
2. Zhang, S. *et al.* Recent progress of porcine milk components and mammary gland function. *J. Anim. Sci. Biotechnol.* **9**, 77 (2018).
3. Hurley, W. L. Composition of sow colostrum and milk. in *The gestating and lactating sow* (ed. Farmer, C.) 193–230 (Wageningen Academic Publishers, The Netherlands, 2015). doi:10.3920/978-90-8686-803-2_9.
4. J. Csapó, T. G. Martin, Z. S. Csapo-Kiss, & Z. Hazas. Protein, fats, vitamin and mineral concentrations in porcine colostrum and milk from parturition to 60 days. *Int. Dairy J.* **6**, 881–902 (1996).

Supplementary Table 3. Differentially expressed genes in small intestine, colon, liver and brain. Group differences were evaluated using DeSeq2 followed by Benjamini & Hochberg FDR correction with 1.5-fold change difference as cutoff.

Small intestine				
**ENSEMBL Gene ID**	**Gene Name**	**Base Mean**	**Log2 Fold Change**	**Adjusted *p*-value**
ALAC ILA producer vs. WPI *(WPI as a reference for log2 fold change)*				
ENSSSCG00000009129	TRAF interacting protein with forkhead associated domain (TIFA)	88.12	0.81	0.013
ALAC ILA nonproducer vs. WPI *(WPI as a reference for log2 fold change)*				
ENSSSCG00000015545	Glutamate-ammonia ligase (GLUL)	531.44	-0.91	0.034
ALAC ILA producer vs. ILA nonproducer *(ILA nonproducer as a reference for log2 fold change)*				
ENSSSCG00000002737	Carbohydrate sulfotransferase 4 (CHST4)	133.83	0.81	0.046
ENSSSCG00000005943	ST3 beta-galactoside alpha-2,3-sialyltransferase 1(ST3GAL1)	365.91	1.66	0.001
ENSSSCG00000006172	Peptidase inhibitor 15 (PI15)	59.26	-0.68	0.036
ENSSSCG00000011928	Coiled-coil domain containing 80 (CCDC80)	76.36	-0.72	0.001
ENSSSCG00000012006	ATP binding cassette subfamily C member 13 (ABCC13)	76.98	0.77	0.036
ENSSSCG00000014310	C-X-C motif chemokine ligand 14(CXCL14)	165.67	-0.80	0.001
ENSSSCG00000015326	Collagen type I alpha 2 chain (COL1A2)	860.73	-0.68	<0.001
ENSSSCG00000016034	Collagen type III alpha 1 chain (COL3A1)	917.89	-0.60	0.012
ENSSSCG00000036135	Collagen type I alpha 1 chain (COL1A1)	798.85	-0.69	<0.001
ENSSSCG00000037697	matrix Gla protein (MGP)	104.76	-1.14	<0.001
ENSSSCG00000049683	Uncharacterized LOC102165634 (LOC102165634)	370.8	-0.67	0.039

Colon				
**ENSEMBL Gene ID**	**Gene Name**	**Base Mean**	**Log2 Fold Change**	**Adjusted *p*-value**
ALAC vs. WPI *(WPI as a reference for log2 fold change)*				
ENSSSCG00000013393	Spondin 1 (SPON1)	51.61	-0.64	0.013
ALAC ILA producer vs. WPI *(WPI as a reference for log2 fold change)*				
ENSSSCG00000003105	Solute carrier family 1 member 5 (SLC1A5)	99.74	-1.06	0.014
ENSSSCG00000003145	Fucosyltransferase 2 (FUT2)	59.50	-0.86	0.046
ENSSSCG00000004542	Asparaginyl-tRNA synthetase 1 (NARS1)	369.95	-0.61	0.035
ENSSSCG00000005601	Heat shock protein family A (Hsp70) member 5 (HSPA5)	878.78	-0.73	0.035
ENSSSCG00000005724	Senataxin(SETX)	229.79	0.73	0.002
ENSSSCG00000008648	Radical S-adenosyl methionine domain containing 2 (RSAD2)	211.26	0.93	0.033
ENSSSCG00000009393	EBP like(EBPL)	46.04	-1.06	0.003
ENSSSCG00000010451	Interferon induced protein with tetratricopeptide repeats 2 (IFIT2)	94.62	1.55	0.014
ENSSSCG00000010452	Interferon-induced protein with tetratricopeptide repeats 1 (IFIT1)	137.68	1.33	0.033
ENSSSCG00000011928	Coiled-coil domain containing 80 (CCDC80)	97.07	-0.69	0.011
ENSSSCG00000012076	Myxovirus (influenza virus) resistance 2 (mouse) (MX2)	286.12	0.90	0.021
ENSSSCG00000013393	Spondin 1 (SPON1)	54.44	-0.72	0.017
ENSSSCG00000014310	C-X-C motif chemokine ligand 14 (CXCL14)	244.44	-1.04	0.008
ENSSSCG00000014988	Matrix metallopeptidase 13 (MMP13)	70.97	-0.86	0.035

ENSSSCG00000015071	SIK family kinase 3 (SIK3)	30.79	0.75	0.045
ENSSSCG00000015897	Interferon induced with helicase C domain 1 (IFIH1)	425.09	0.61	0.012
ENSSSCG00000017146	E3 ubiquitin-protein ligase RNF213 (A0A287A1Y8_PIG)	51.30	0.86	0.03
ENSSSCG00000021036	RAB3B, member RAS oncogene family (RAB3B)	29.17	-0.88	0.035
ENSSSCG00000022986	Coiled-coil serine rich protein 1 (CCSER1)	59.64	0.67	0.021
ENSSSCG00000025924	Insulin like growth factor binding protein 5 (IGFBP5)	148.31	-0.77	0.003
ENSSSCG00000027372	Sterile alpha motif domain-containing protein 9 (LOC100519098)	207.05	0.69	0.017
ENSSSCG00000027660	Interferon induced protein 44 like (IFI44L)	150.95	0.59	0.011
ENSSSCG00000030408	RNA sensor RIG-I (RIGI)	201.53	0.99	0.006
ENSSSCG00000032374	Sulfotransferase family cytosolic 1B member 1 (LOC100624541)	278.96	0.71	0.003
ENSSSCG00000033089	Interferon-induced very large GTPase 1-like (LOC110255360)	30.55	1.19	0.012
ENSSSCG00000034827	Calcineurin like EF-hand protein 2 (CHP2)	1110.12	0.61	0.035
ENSSSCG00000034994	A-kinase anchoring protein 1 (AKAP1)	48.97	0.64	0.043
ENSSSCG00000036639	Stomatin (STOM)	232.87	-0.79	0.033
ENSSSCG00000037645	Coactosin like F-actin binding protein 1 (COTL1)	131.01	-1.11	0.017
ENSSSCG00000055651	Complement C1s (C1S)	120.16	-0.93	0.011
ENSSSCG00000056284	XIAP associated factor 1 (XAF1)	25.67	0.97	0.012
ENSSSCG00000061268	Interferon induced protein with tetratricopeptide repeats 3 (IFIT3)	52.57	1.81	<0.001
ALAC ILA producer vs. ILA nonproducer *(ILA nonproducer as a reference for log2 fold change)*				
ENSSSCG00000000253	Keratin 18 (KRT18)	103.18	-0.71	0.043
ENSSSCG00000000377	Proliferation-associated 2G4 (PA2G4)	550.79	-0.59	0.006
ENSSSCG00000002350	Mitotic deacetylase associated SANT domain protein (MIDEAS)	38.87	0.87	0.032
ENSSSCG00000003105	Solute carrier family 1 member 5 (SLC1A5)	78.14	-0.65	0.042
ENSSSCG00000003145	Fucosyltransferase 2 (FUT2)	68.23	-0.73	0.024
ENSSSCG00000003189	Protein arginine methyltransferase 1 (PRMT1)	85.77	-0.83	0.008
ENSSSCG00000003663	Hippocalcin like 1 (HPCAL1)	115.33	-0.73	0.015
ENSSSCG00000003687	Erythrocyte membrane protein band 4.1 like 3 (EPB41L3)	33.85	1.01	0.011
ENSSSCG00000004233	Sphingomyelin phosphodiesterase acid like 3A (SMPDL3A)	70.08	0.73	0.02
ENSSSCG00000004254	Discoidin, CUB and LCCL domain containing 1 (DCBLD1)	74.23	-0.65	0.043
ENSSSCG00000004542	Asparaginyl-tRNA synthetase 1 (NARS1)	352.83	-0.60	0.026
ENSSSCG00000004565	Carbonic anhydrase 12 (CA12)	61.01	0.77	0.026
ENSSSCG00000005269	Transient receptor potential cation channel subfamily M member 6 (TRPM6)	72.70	0.70	0.047
ENSSSCG00000005596	Actin related protein 2/3 complex subunit 5 like (ARPC5L)	122.98	-0.64	0.006
ENSSSCG00000005601	Heat shock protein family A (Hsp70) member 5 (HSPA5)	957.91	-0.86	<0.001
ENSSSCG00000005724	Senataxin (SETX)	241.65	0.83	<0.001
ENSSSCG00000006126	2,4-Dienoyl-CoA reductase 1 (DECR1)	63.14	0.61	0.045
ENSSSCG00000006337	Hydroxysteroid 17-beta dehydrogenase 7 (HSD17B7)	74.65	-0.72	0.013
ENSSSCG00000007275	RALY heterogeneous nuclear ribonucleoprotein (RALY)	112.19	-0.66	0.008
ENSSSCG00000007485	Brain enriched myelin associated protein 1 (BCAS1)	98.22	-0.60	0.044
ENSSSCG00000007507	Phosphoenolpyruvate carboxykinase 1 (PCK1)	69.32	1.63	0.018

ENSSSCG00000007508	Z-DNA binding protein 1 (ZBP1)	49.23	1.18	0.002
ENSSSCG00000008496	Eukaryotic translation initiation factor 2 alpha kinase 2 (EIF2AK2)	901.25	0.59	0.015
ENSSSCG00000008633	Protein disulfide isomerase family A member 6 (PDIA6)	384.06	-0.62	0.012
ENSSSCG00000008647	Cytidine/uridine monophosphate kinase 2 (CMPK2)	117.28	0.64	0.027
ENSSSCG00000008648	Radical S-adenosyl methionine domain containing 2 (RSAD2)	205.18	1.96	0.001
ENSSSCG00000008803	ATPase phospholipid transporting 8A1 (ATP8A1)	65.24	1.00	0.009
ENSSSCG00000009240	Placenta associated 8 (PLAC8)	52.61	1.39	0.006
ENSSSCG00000009408	Leucine rich repeats and calponin homology domain containing 1 (LRCH1)	44.56	0.73	0.043
ENSSSCG00000010212	Ankyrin 3 (ANK3)	171.43	0.64	0.045
ENSSSCG00000010370	Annexin A8 (ANXA8)	45.85	-1.27	0.004
ENSSSCG00000010403	Membrane associated ring-CH-type finger 8 (MARCHF8)	39.07	0.85	0.02
ENSSSCG00000010437	3'-Phosphoadenosine 5'-phosphosulfate synthase 2 (PAPSS2)	199.29	0.90	0.004
ENSSSCG00000010451	Interferon induced protein with tetratricopeptide repeats 2 (IFIT2)	96.99	1.61	0.028
ENSSSCG00000010452	Interferon-induced protein with tetratricopeptide repeats 1 (IFIT1)	149.11	1.91	0.002
ENSSSCG00000010568	Nucleophosmin/nucleoplasmin 3 (NPM3)	72.31	-0.85	0.048
ENSSSCG00000011239	Tetratricopeptide repeat and ankyrin repeat containing 1 (TRANK1)	48.09	0.97	0.006
ENSSSCG00000011874	Poly(ADP-ribose) polymerase family member 14 (PARP14)	545.94	0.60	0.022
ENSSSCG00000011928	Coiled-coil domain containing 80 (CCDC80)	88.34	-0.78	0.035
ENSSSCG00000012074	Beta-secretase 2 (BACE2)	73.73	-0.61	0.042
ENSSSCG00000012076	Myxovirus (influenza virus) resistance 2 (mouse) (MX2)	294.99	1.32	0.015
ENSSSCG00000012257	Monoamine oxidase A (MAOA)	113.87	0.67	0.024
ENSSSCG00000013380	Nucleobindin 2 (NUCB2)	78.23	-0.82	0.024
ENSSSCG00000013551	Complement C3 (C3)	278.50	-0.84	0.004
ENSSSCG00000014156	Arrestin domain containing 3 (ARRDC3)	160.86	0.66	0.006
ENSSSCG00000014310	C-X-C motif chemokine ligand 14 (CXCL14)	266.62	-0.98	0.001
ENSSSCG00000015106	Hypoxia up-regulated 1 (HYOU1)	240.35	-0.64	0.007
ENSSSCG00000015250	ADAM metallopeptidase with thrombospondin type 1 motif 15 (ADAMTS15)	71.47	-1.16	0.007
ENSSSCG00000015340	Asparagine synthetase (glutamine-hydrolyzing) (ASNS)	33.01	-1.06	0.011
ENSSSCG00000015390	ATP-binding cassette, sub-family B (MDR/TAP), member 1 (ABCB1)	59.57	1.94	<0.001
ENSSSCG00000015897	Interferon induced with helicase C domain 1 (IFIH1)	417.87	1.02	<0.001
ENSSSCG00000016057	Signal transducer and activator of transcription 1 (STAT1)	651.91	0.63	0.014
ENSSSCG00000016114	Family with sequence similarity 117 member B (FAM117B)	80.67	1.33	<0.001
ENSSSCG00000016263	Nuclear body protein SP140-like protein (LOC100517129)	104.78	0.77	0.002
ENSSSCG00000016609	Solute carrier family 13 member 1 (SLC13A1)	51.18	0.76	0.018
ENSSSCG00000017104	NOP2/Sun RNA methyltransferase 2 (NSUN2)	200.93	-0.60	0.011
ENSSSCG00000017798	Transmembrane and immunoglobulin domain containing 1 (TMIGD1)	119.41	0.82	0.036
ENSSSCG00000017835	Clustered mitochondria homolog (CLUH)	137.58	-0.74	0.002
ENSSSCG00000017957	Eukaryotic translation initiation factor 4A1 (EIF4A1)	79.55	-0.71	0.02
ENSSSCG00000021408	Transketolase(TKT)	191.01	-0.59	0.02
ENSSSCG00000023434	Protein phosphatase, Mg2+/Mn2+ dependent 1L (PPM1L)	198.00	0.66	0.005

ENSSSCG00000026454	Phorbol-12-myristate-13-acetate-induced protein 1 (PMAIP1)	47.80	1.07	0.008
ENSSSCG00000026605	Bactericidal permeability increasing protein (BPI)	286.99	-0.93	0.004
ENSSSCG00000026697	Bridge-like lipid transfer protein family member 3B (BLTP3B)	162.01	0.68	0.008
ENSSSCG00000026748	Polo-like kinase 1(PLK1)	73.48	-0.68	0.042
ENSSSCG00000027070	Ectonucleoside triphosphate diphosphohydrolase 6 (ENTPD6)	49.29	-0.70	0.025
ENSSSCG00000027372	Sterile alpha motif domain-containing protein 9 (LOC100519098)	220.64	0.67	0.004
ENSSSCG00000027709	Poly(ADP-ribose) polymerase family member 9 (PARP9)	70.58	0.82	0.004
ENSSSCG00000027894	Family with sequence similarity 76 member A (FAM76A)	43.41	0.69	0.042
ENSSSCG00000028019	Leucine rich repeat containing 59 (LRRC59)	121.57	-0.84	0.012
ENSSSCG00000029275	PPARG coactivator 1 alpha (PPARGC1A)	87.62	0.86	0.005
ENSSSCG00000030108	Zinc finger NFX1-type containing 1 (ZNFX1)	163.05	0.76	0.018
ENSSSCG00000030408	RNA sensor RIG-I (RIGI)	206.22	1.47	<0.001
ENSSSCG00000030548	HECT and RLD domain containing E3 ubiquitin protein ligase 5 (HERC5)	152.88	0.75	0.02
ENSSSCG00000031262	Thioredoxin interacting protein (TXNIP)	1771.39	0.97	<0.001
ENSSSCG00000032996	Solute carrier family 7 member 5 (SLC7A5)	57.83	-0.77	0.019
ENSSSCG00000036033	Thyroid hormone receptor beta (THRB)	97.33	0.87	0.007
ENSSSCG00000036790	A-kinase anchoring protein 7 (AKAP7)	87.13	0.82	0.003
ENSSSCG00000036883	Fatty acid binding protein 3 (FABP3)	312.77	-0.71	0.017
ENSSSCG00000037637	Gamma-glutamyl hydrolase (GGH)	38.57	0.95	0.012
ENSSSCG00000037645	Coactosin like F-actin binding protein 1 (COTL1)	115.69	-1.16	<0.001
ENSSSCG00000038182	Double-headed protease inhibitor, submandibular gland-like (LOC100739218)	342.31	-1.23	0.038
ENSSSCG00000039341	Sulfotransferase family 1E, estrogen-preferring, member 1 (SULT1E1)	148.15	0.89	0.016
ENSSSCG00000040735	Dimethylarginine dimethylaminohydrolase 1 (DDAH1)	96.13	-0.66	0.013
ENSSSCG00000040770	Carbonic anhydrase 1 (CA1)	5267.28	0.82	0.013
ENSSSCG00000040947	Regenerating family member 4 (REG4)	790.74	-1.13	0.042
ENSSSCG00000052127	SP140 nuclear body protein (SP140)	165.37	0.69	0.016
ENSSSCG00000056284	XIAP associated factor 1 (XAF1)	28.26	1.11	0.011
ENSSSCG00000057184	Centromere protein W (CENPW)	43.41	-0.74	0.043
ENSSSCG00000059584	RNA polymerase II, I and III subunit L (POLR2L)	131.32	-0.65	0.013
ENSSSCG00000061023	Receptor transporter protein 4 (RTP4)	163.45	1.17	0.003
ENSSSCG00000061268	Interferon induced protein with tetratricopeptide repeats 3 (IFIT3)	59.94	1.86	0.011

Liver

ENSEMBL Gene ID	Gene Name	Base Mean	Log2 Fold Change	Adjusted *p*-value
ALAC vs. WPI *(WPI as a reference for log2 fold change)*				
ENSSSCG00000002623	Glutathione S-transferase alpha M14 (LOC106504562)	922.85	0.85	0.021
ALAC ILA nonproducer vs. WPI *(WPI as a reference for log2 fold change)*				
ENSSSCG00000001906	Cytochrome P450 family 1 subfamily A member 1 (CYP1A1)	107.13	2.22	0.023
ENSSSCG00000002623	Glutathione S-transferase alpha M14 (LOC106504562)	862.75	1.19	<0.001
ALAC ILA producer vs. ILA nonproducer *(ILA nonproducer as a reference for log2 fold change)*				
ENSSSCG00000000259	Cysteine sulfinic acid decarboxylase (CSAD)	243.82	0.74	0.006

ENSSSCG00000000401	Glutaminase 2 (GLS2)	148.01	0.87	0.004
ENSSSCG00000000849	Heat shock protein 90 beta family member 1 (HSP90B1)	2718.11	-0.80	0.013
ENSSSCG00000004510	Acetyl-CoA acyltransferase 2 (ACAA2)	462.74	0.87	<0.001
ENSSSCG00000006238	Cytochrome P450 family 7 subfamily A member 1 (CYP7A1)	57.94	2.39	<0.001
ENSSSCG00000008484	Serine and arginine rich splicing factor 7 (SRSF7)	131.35	-0.68	0.01
ENSSSCG00000008648	Radical S-adenosyl methionine domain containing 2 (RSAD2)	155.72	1.58	<0.001
ENSSSCG00000009184	Alcohol dehydrogenase 4 (class II), pi polypeptide (ADH4)	3961.78	-1.11	<0.001
ENSSSCG00000009240	Placenta associated 8 (PLAC8)	96.12	0.62	0.027
ENSSSCG00000009720	DExD/H-box helicase 60 (DDX60)	88.51	0.88	0.01
ENSSSCG00000009881	2'-5'-Oligoadenylate synthetase 2 (OAS2)	384.14	1.06	0.007
ENSSSCG00000010452	Interferon-induced protein with tetratricopeptide repeats 1 (IFIT1)	439.58	1.16	0.001
ENSSSCG00000012077	MX dynamin like GTPase 1 (MX1)	478.61	0.85	0.014
ENSSSCG00000013380	Nucleobindin 2 (NUCB2)	46.44	-1.41	0.005
ENSSSCG00000013665	Complement C3 (LOC100517145)	386.37	0.59	0.005
ENSSSCG00000015326	Collagen type I alpha 2 chain (COL1A2)	276.05	-1.69	<0.001
ENSSSCG00000015453	Protein disulfide isomerase family A member 4 (PDIA4)	524.16	-0.97	<0.001
ENSSSCG00000015735	Protein tyrosine phosphatase non-receptor type 18 (PTPN18)	84.73	-0.78	0.007
ENSSSCG00000016034	Collagen type III alpha 1 chain (COL3A1)	392.90	-1.02	<0.001
ENSSSCG00000016057	Signal transducer and activator of transcription 1 (STAT1)	690.03	0.67	0.005
ENSSSCG00000016652	Leucine rich repeat neuronal 3 (LRRN3)	68.86	0.89	0.005
ENSSSCG00000021712	HECT and RLD domain containing E3 ubiquitin protein ligase family member 6 (HERC6)	98.22	0.83	0.01
ENSSSCG00000023653	GLIS family zinc finger 2 (GLIS2)	96.36	-0.60	0.034
ENSSSCG00000025788	Ectonucleotide pyrophosphatase/phosphodiesterase 4 (ENPP4)	56.97	-0.93	0.01
ENSSSCG00000030108	Zinc finger NFX1-type containing 1 (ZNFX1)	204.52	0.84	0.004
ENSSSCG00000030408	RNA sensor RIG-I (RIGI)	242.03	1.19	<0.001
ENSSSCG00000030548	HECT and RLD domain containing E3 ubiquitin protein ligase 5 (HERC5)	62.10	0.99	0.018
ENSSSCG00000034735	UDP-glucuronosyltransferase 2B31-like (LOC100623504)	1143.17	0.65	0.042
ENSSSCG00000036135	Collagen type I alpha 1 chain (COL1A1)	318.08	-1.48	<0.001
ENSSSCG00000036227	Zinc finger protein 277-like (A0A287BQQ0_PIG)	222.76	0.84	<0.001
ENSSSCG00000036785	Myotubularin related protein 7 (MTMR7)	57.88	1.53	0.002
ENSSSCG00000055280	Uncharacterized LOC106504881 (LOC106504881)	118.22	1.14	<0.001
ENSSSCG00000061268	Interferon induced protein with tetratricopeptide repeats 3(IFIT3)	141.47	1.06	0.007

Brain

ENSEMBL Gene ID	Gene Name	Base Mean	Log2 Fold Change	Adjusted *p*-value
ALAC ILA producer vs. WPI *(WPI as a reference for log2 fold change)*				
ENSSSCG00000009330	Arachidonate 5-lipoxygenase activating protein (ALOX5AP)	27.35	-0.62	0.013
ALAC ILA producer vs. WPI *(WPI as a reference for log2 fold change)*				
ENSSSCG00000014725	Hemoglobin, beta (HBB)	3336.86	0.73	0.023
ALAC ILA producer vs. ILA nonproducer *(ILA nonproducer as a reference for log2 fold change)*				
ENSSSCG00000001438	PBX homeobox 2 (PBX2)	23.54	0.68	0.005

ENSSSCG00000006555	Cilia and flagella associated protein 141 (CFAP141)	2.48	-1.38	0.028
ENSSSCG00000008648	Radical S-adenosyl methionine domain containing 2 (RSAD2)	12.09	2.52	<0.001
ENSSSCG00000010451	Interferon induced protein with tetratricopeptide repeats 2 (IFIT2)	16.55	0.84	0.013
ENSSSCG00000010452	Interferon-induced protein with tetratricopeptide repeats 1(IFIT1)	54.87	0.87	<0.001
ENSSSCG00000011874	Poly(ADP-ribose) polymerase family member 14 (PARP14)	66.02	0.67	0.006
ENSSSCG00000011876	Deltex E3 ubiquitin ligase 3L (DTX3L)	21.97	0.78	<0.001
ENSSSCG00000012076	Myxovirus (influenza virus) resistance 2 (mouse) (MX2)	68.41	1.20	<0.001
ENSSSCG00000014725	Hemoglobin, beta (HBB)	370.67	-0.68	0.004
ENSSSCG00000015897	Interferon induced with helicase C domain 1 (IFIH1)	41.12	0.68	0.001
ENSSSCG00000016502	Poly(ADP-ribose) polymerase family member 12 (PARP12)	11.08	1.09	<0.001
ENSSSCG00000020906	TNF superfamily member 10 (TNFSF10)	56.32	0.88	<0.001
ENSSSCG00000022195	Zinc finger with KRAB and SCAN domains 7 (ZKSCAN7)	2.77	-1.73	0.001
ENSSSCG00000027660	Interferon induced protein 44 like (IFI44L)	37.69	0.87	<0.001
ENSSSCG00000032474	C-X-C motif chemokine ligand 10 (CXCL10)	20.40	1.09	<0.001
ENSSSCG00000033089	Interferon-induced very large GTPase 1-like (LOC110255360)	7.65	0.91	0.023
ENSSSCG00000034743	Centrin 4 (CETN4)	113.15	-0.63	<0.001
ENSSSCG00000036135	Collagen type I alpha 1 chain (COL1A1)	52.09	-0.76	0.036
ENSSSCG00000037572	Epithelial stromal interaction 1 (EPSTI1)	31.02	0.98	<0.001
ENSSSCG00000061268	Interferon induced protein with tetratricopeptide repeats 3 (IFIT3)	23.85	0.82	0.001
